# Multi-product biorefinery from *Arthrospira platensis* biomass as feedstock for bioethanol and lactic acid production

**DOI:** 10.1038/s41598-021-97803-5

**Published:** 2021-09-29

**Authors:** Diego A. Esquivel-Hernández, Anna Pennacchio, Mario A. Torres-Acosta, Roberto Parra-Saldívar, Luciana Porto de Souza Vandenberghe, Vincenza Faraco

**Affiliations:** 1 Escuela de Ingenieria y Ciencias, Tecnologico de Monterrey, Campus Monterrey, Ave. Eugenio Garza Sada 2501, 64849 Monterrey, NL Mexico; 2 Department of Chemical Sciences, University of Naples “Federico II”, Complesso Universitario Monte S. Angelo, Via Cintia 4, 80126 Naples, Italy; 3 Department of Biochemical Engineering, The Advance Centre for Biochemical Engineering, University College London, London, WC1E 6BT UK; 4 Department of Bioprocess Engineering and Biotechnology, Federal University of Paraná, Coronel Francisco H. dos Santos Avenue, 210, Curitiba, 81531-980 Brazil; 5 Present Address: Departamento de Microbiologia Molecular, Instituto de Biotecnologia, Universidad Nacional Autónoma de México, Ave. Universidad 2001, 62210 Cuernavaca, Morelos Mexico; 6 Present Address: Departamento de Biología Celular, Facultad de Ciencias, Universidad Nacional Autónoma de México, Circuito Exterior s/n, 04510 Mexico City, Mexico

**Keywords:** Biotechnology, Microbiology

## Abstract

With the aim to reach the maximum recovery of bulk and specialty bioproducts while minimizing waste generation, a multi-product biorefinery for ethanol and lactic acid production from the biomass of cyanobacterium *Arthrospira platensis* was investigated. Therefore, the residual biomass resulting from different pretreatments consisting of supercritical fluid extraction (SF) and microwave assisted extraction with non-polar (MN) and polar solvents (MP), previously applied on *A. platensis* to extract bioactive metabolites, was further valorized. In particular, it was used as a substrate for fermentation with *Saccharomyces cerevisiae* LPB-287 and *Lactobacillus acidophilus* ATCC 43121 to produce bioethanol (BE) and lactic acid (LA), respectively. The maximum concentrations achieved were 3.02 ± 0.07 g/L of BE by the MN process at 120 rpm 30 °C, and 9.67 ± 0.05 g/L of LA by the SF process at 120 rpm 37 °C. An economic analysis of BE and LA production was carried out to elucidate the impact of fermentation scale, fermenter costs, production titer, fermentation time and cyanobacterial biomass production cost. The results indicated that the critical variables are fermenter scale, equipment cost, and product titer; time process was analyzed but was not critical. As scale increased, costs tended to stabilize, but also more product was generated, which causes production costs per unit of product to sharply decrease. The median value of production cost was US$ 1.27 and US$ 0.39, for BE and LA, respectively, supporting the concept of cyanobacterium biomass being used for fermentation and subsequent extraction to obtain ethanol and lactic acid as end products from *A. platensis*.

## Introduction

The cyanobacterium *Arthrospira platensis* is an outstanding sunlight-driven cytoplasm capable to convert carbon dioxide (CO_2_) into high value metabolites (HVM)^1^. This photosynthetic cyanobacterium have received much attention for its HVM such as α-tocopherol, β-carotene, lutein and γ-linolenic acid^2,3^ as well for its bioactive effects (e.g. antifungal, antibacterial, antiviral, antimycotic, cytotoxicity, multi-drug resistance reversers and immunosuppressive actions)^4–6^. Also, cyanobacteria consume CO_2_ as carbon source, use sunlight for energy and have high productivities^7^. Based on the above, *A. platensis* is a promising renewable feedstock for biorefineries. For example, by producing both bulk and HVM from *A. platensis* the total revenue from cyanobacteria can increase. Production of bulk commodities (e.g., biofuels, bioplastics) can become economically convenient^7–9^ within a multi-product biorefinery plant^10–13^. Recent advancements in this direction has spotted considerable benefits and its entanglement in the circular economy^14^.

Moreover and by 2026, the global market value for *A. platensis* is estimated in 779 USD million with a compound annual growth rate (CAGR) of 10.6% between 2019 and 2026^15^. Therefore, its cultivation can be used in a money-spinning strategy through the development of a multi-product biorefinery that aims the maximum recovery of bulk and specialty bioproducts while minimizing waste generation^16^.

Bioethanol (BE) is an environmentally friendly biofuel that has been largely commercialized and tested^17^. Nevertheless, the first and second generation of biofuels cannot satisfy global demand in sustainable practices^18^. The kind of feedstock is the major disadvantage for these types of biofuels. The third generation of biofuels comes from non-food feedstocks such as microalgae, cyanobacteria and macroalgae biomass. which have shown a great potential for the production of biofuels^19^. Use of these non-food feedstocks can be even more valorized using most of both the components of the biomasses and byproducts of their conversion processes in order to minimize waste generation while maximizing value recovery aiming at the zero-waste biorefinery concept.

Furthermore, the challenges ahead for third generation biofuel production are the low yield of biomass production with some microalgal species such as *Chlorella*^20^ and *Haematococcus*^21^, the harvesting of the biomass and related high energy inputs. All these bottlenecks can be overcome by application of the cyanobacterium *A. platensis*.

The low availability of sustainable feedstocks applied to bioplastic production, which represents another a strategic market. In 2014, the global bioplastic production strength was 1.7 million tons, which can rise up to 9 million tons by 2021. In this sector, polylactic acid (PLA) is the dominant biopolymer for the future^22^. Plants can serve as feedstock for the production of LA by microbial fermentation^23^. However, the feedstock adopted for the industrial production of LA pose economic problems with commercial substrates (i.e. glucose, lactose, starch) or ethical problems in case of first-generation feedstock (i.e. potato, wheat and rice)^24^. Moreover, the use of whey milk (as second-generation feedstock) deals with several drawbacks. For example, the high water content and storage problems due to its susceptibility to bacterial and fungal spoilage; the use of highly expensive techniques of reverse osmosis and ultrafiltration as a pretreatment of whey milk^25^, and the need of enrichment of whey with nitrogen sources such as yeast extract, peptone, among others to improve LA production^26^. Thus, the entire operation was more expensive and unfeasible for its application on an industrial scale^27^. In this way, cyanobacteria and algae biomass can be catalogued as the most promising bio-feedstock for renewable LA production^28^.

**Figure 1:**
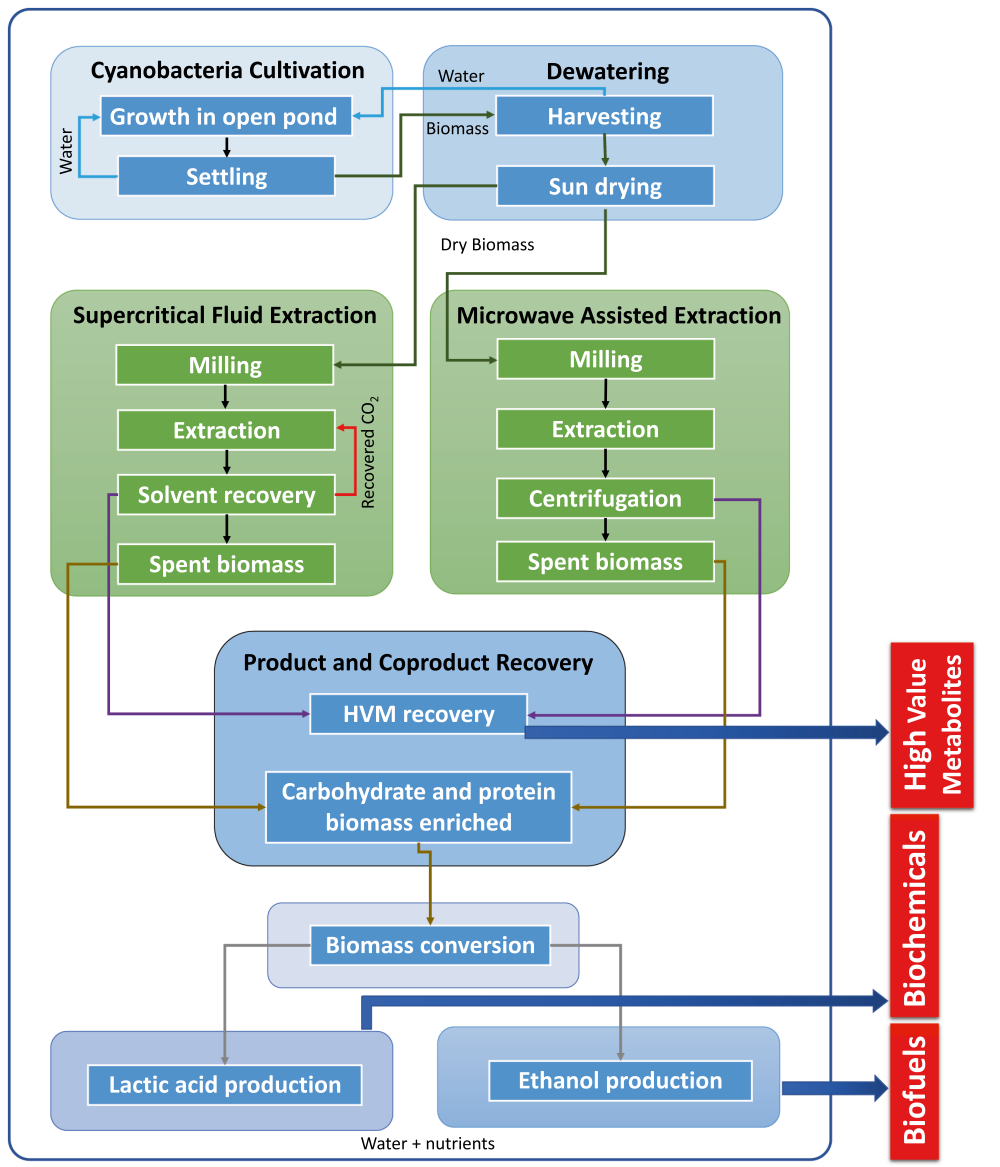
Cyanobacteria biomass and by-products conversion into biofuels, biochemicals and HVM through green extraction technologies and fermentation processes in the frame of a multi-product biorefinery.

Research and development of biorefineries based on *A. platensis* biomass is scarce, with just few reports on co-production of energy-based products and simultaneous production of biodiesel, specialty bioproducts coupled with wastewater bioremediation, polysaccharides extraction through green processes and re-use of residual fractions from protein extraction^29–33^. Hence, the goal of this paper is to evaluate the development of a multi-product biorefinery from *A. platensis* biomass for the production of bulk bioproducts [e.g., BE, lactic acid (LA)] and HVM, with evaluation of fermentation process parameters for the production of LA and BE (Fig. 1). Therefore, the residual biomass resulting from pretreatments consisting of supercritical fluid extraction (SF) and microwave assisted extraction with non-polar (MN) and polar solvents (MP), previously applied on *A. platensis* to extract bioactive metabolites^34,35^ (Supplementary Table S1) was further valorized in this work. In particular, it was used as a substrate for fermentation with *Saccharomyces cerevisiae* LPB-287 and *Lactobacillus acidophilus* ATCC 43121 to produce bioethanol (BE) and lactic acid (LA), respectively. Additionally, an economic analysis was performed to elucidate the impact of different production scales, material and fermenters costs, potential optimization of fermentation titer and process duration on the production costs of ethanol and lactic acid.

## Results

A multi-product biorefinery for BE and LA production from the biomass of cyanobacterium *A. platensis* was investigated. The lack of lignin in the cell wall of *A. platensis* entails milder pre-treatments for releasing the fermentable sugars than lignocellulosic biomasses, and overcomes the problems related to pretreatments needed with other feedstock^36^. Different mild pretreatments consisting of supercritical fluid extraction (SF) and microwave assisted extraction with non-polar (MN) and polar solvents (MP) were tested individually on cyanobacterial biomass to obtain HVM and the resulting residual biomass was used as a substrate for fermentation with *Saccharomyces cerevisiae* LPB-287 and *Lactobacillus acidophilus* ATCC 43121 to produce BE and LA, respectively.

### Influence of supercritical fluid extraction (SF) pretreatment of cyanobacterial biomass on ethanol and lactic acid production

**Figure 2:**
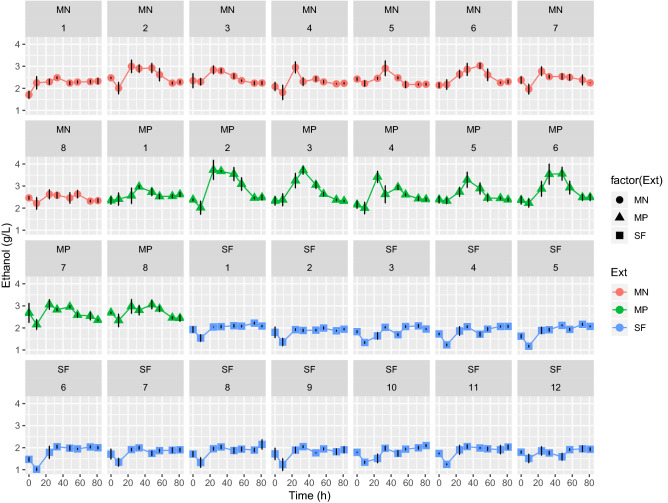
Time course of concentration of BE in the fermentation process by *Saccharomyces cerevisiae LPB-287* with depleted cyanobacteria biomass from SF, MN and MP pretreatments. *(factor(Ext) refers to the type of pretreatment respectively, n = 3; time of fermentation 90 h).

**Figure 3:**
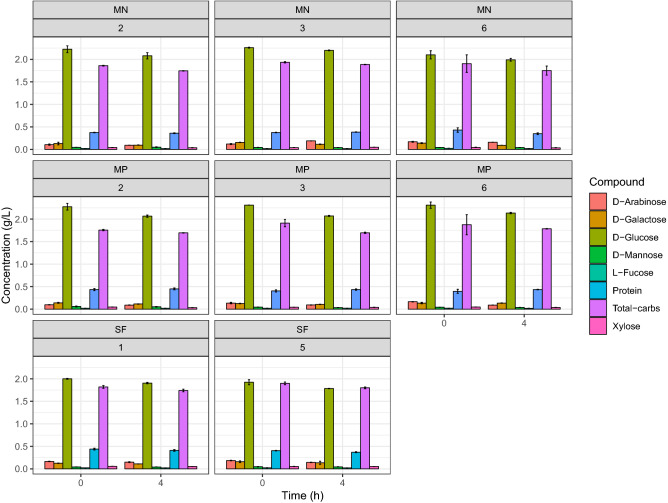
Total protein and monosaccharides concentration profile in the fermentation process by *Saccharomyces cerevisiae LPB-287* with cyanobacteria from SF, MN and MP pretreatments of selected samples. *(n = 2, time of sampling 0 and 4 h).

A Plackett–Burman design was used to select different factors influencing SF process of cyanobacterial biomass that was used as substrate for BE production by *S. cerevisiae* LPB-287 after the extraction of HVM. The investigated factors included pressure, temperature, co-solvent (ethanol), dispersant agent (glass pearls), and static and dynamic extraction. As shown in Fig. 2, no significant differences were observed in BE production that reached 2.0 ± 0.08 g/L with highest yield of 0.20 (% gEthanol/gCyanobacteria) (Supplementary Fig. S1). As shown in Supplementary Fig. S2, SF pretreatment promoted lower concentrations of reducing sugars content when compared with the control biomass. This can be due to the water content in BE for SF. The highest content of reducing sugars was obtained in the experiments SF8 CS = 11 g/min, P = 450 bar, T = 40 °C and 1 CS = 4 g/min, P = 450 bar and T = 40 °C Supplementary Table S2a. Monosaccharides and protein content of *S. cerevisiae* fermentation of SF pretreated *A. platensis* biomass was performed. For SF, the experiments SF1 CS = 4 g/min, P = 450 bar, T = 40 °C and SF5 CS = 11 g/min, P = 150 bar, T = 40°, were selected respectively, at two different times based on their high content of BE. The profile of monosaccharides showed a higher content of d-glucose (2.01 ± 0.08, g/L) for SF, in comparison with the other monosaccharides such as d-galactose, d-mannose, d-arabinose, l-fucose and xylose (Fig. 3). In terms of protein content, SF achieved 0.47 ± 0.05 g/L and no changes were observed.

*Saccharomyces cerevisiae* ferments glucose, which represents the dominant sugar in almost all hydrolysates from biomass used as carbon source for BE production^37^. Also, mannose and fructose are two isomers of glucose present in the biomass hydrolysates that can be fermented by *S. cerevisiae*. As far as we know, the yeast capable of fermenting glucose and that can also ferment fructose and mannose, is known as the Kluyver rule^38^. According to this rule, we assume that the strain *S. cerevisiae* LPB-287 can ferment mannose, but its consumption is mediated by the kinetics of mixed-substrate utilization because both compete for the same hexose transporters^39^. Furthermore, in our experiments we detected galactose (Fig. 3) but the glucose concentration caused a complete repression of galactose metabolism^40^, thus prevented the simultaneous consumption of glucose and galactose. However, sequential consumption of different monosaccharides can represent a problem for BE production, due to the lag phase that separates glucose and galactose consumption in mixed-substrate cultures similar to our experiments. Therefore, this problem needs to be undertaken. Xylose and arabinose cannot be fermented or assimilated by *S. cerevisiae* LPB-87, and thus their concentration does not change along the experiments (Fig. 3). In general, it can be seen that in all cases (SF) there was a strong effect on fermentation mediated by Carbon Catabolite Repression (CCR) because the hexose sugars were rapidly metabolized by *S. cerevisiae*. Besides, our results showed (Fig. 3) that *S. cerevisiae* used pentose sugars when the hexoses were consumed^41^. Kinetics of assimilation of this mixture of sugars has cycles of 24 h for the consumption of hexose and pentose sugars. Hexose sugars are considered to be efficiently fermented to BE, whereas pentose sugars were consumed after the depletion of hexose sugars^42^.

**Figure 4:**
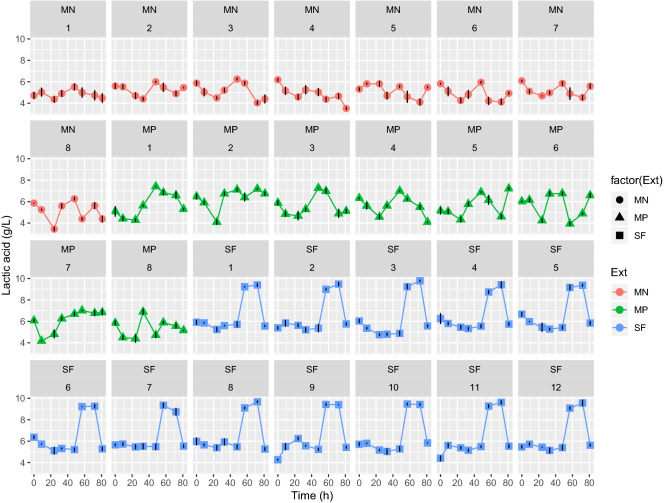
Time course of concentration and yield of LA in the fermentation process by *Lactobacillus acidophilus* ATCC 43121 with cyanobacteria from SF, MN and MP pretreatments. *(factor(Ext) refers to the type of pretreatment respectively, n = 3, time of fermentation 90 h).

Additionally, the depleted biomass was adopted as substrate for LA fermentation by *L*. *acidophilus* ATCC 43121. As shown in Fig. 4, the highest concentrations of LA, 9.67 ± 0.05 and 9.62 ± 0.05 g/L, were achieved with the experiments SF8 and SF11, respectively. In relation to, the LA’s yield for SF, the highest yield (% gLactic acid/gCyanobacteria) was 0.48 in the experiments SF8 CS = 11 g/min, P = 450 bar, T = 40 °C and 11 CS = 11 g/min, P = 150 bar, T = 60 °C (Supplementary Fig. S3). As for the assimilation of cyanobacterial biomass by *L. acidophilus* ATCC 43121, we measured the change in reducing sugars content over time. These results are presented in Supplementary Fig. S4. For SF, all extraction samples evaluated display a decrease in glucose content when compared with the control (Supplementary Fig. S4). This role can be due to metabolism of the genus *Lactobacillus* as the hexose sugars are rapidly metabolized because *L. acidophilus* has a global regulatory system of carbon catabolite repression (CCR), which controls the transport and catabolism of carbohydrates^43,44^. Thus, through this regulatory mechanism *L. acidophilus* cells can coordinate carbohydrate utilization and focus primarily on preferred energy sources^45^.

**Figure 5:**
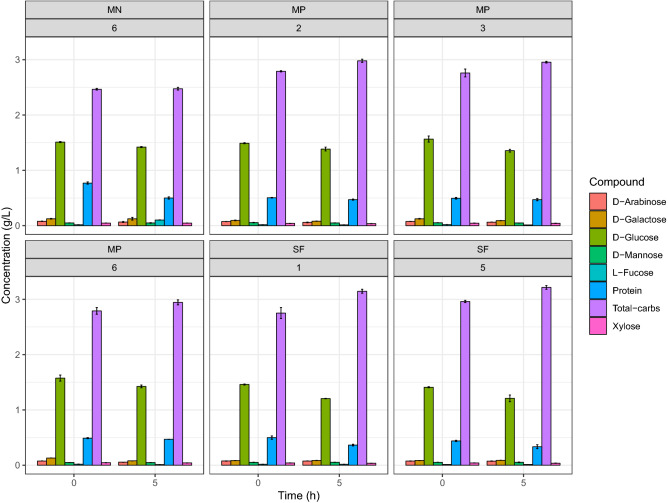
Total protein and monosaccharides concentration profile in the fermentation process by *Lactobacillus acidophilus* ATCC 43121 with cyanobacteria from SF, MN and MP pretreatments of selected samples. *(n = 2, time of sampling 0 and 5 h).

However, the depleted cyanobacterial biomass contained other kind of sugars such as pentose sugars that *L. acidophilus* does not use in presence of hexoses sugars, which are considered to be efficiently fermented into LA^46^. The highest content of reducing sugars was obtained under the experiments SF5 and SF10 (Supplementary Table S2a, Supplementary Fig. S4). The time of course of monosaccharides and protein of the most representative samples of SF, pretreatments in the fermentation by *L. acidophilus* was performed. The experiments SF1 CS = 4 g/min, P = 450 bar, T = 40 °C and SF5 CS = 11 g/min, P = 150 bar, T = 60 °C, were selected respectively, at two different times based on their high content of LA. The profile of monosaccharides revealed a high content of d-glucose (1.46 ± 0.07, g/L) for SF, in comparison with the other monosaccharides such as d-galactose, d-mannose, d-arabinose, l-fucose and xylose (Fig. 5).

### Influence of microwave assisted extraction (MP/MN) pretreatment of cyanobacterial biomass on ethanol and lactic acid production

For microwave assisted extraction pretreatment, polar (MP) and non-polar solvents (MN) were applied as extraction techniques, to obtain substrates that we used as carbon source in BE fermentation by *S. cerevisiae* LPB-287. As shown in Fig. 2, the highest BE concentrations were obtained in the experiment MN6 (3.02 ± 0.07 g/L) and experiment MN2 (2.99 ± 0.07 g/L). Best BE production was reached with MN pretreatment than MP pretreatment. This can be due to the use of non-polar solvents in MN pretreatment with led to higher carbohydrates content substrate with consequent higher BE yield 0.30 (% gEthanol/gCyanobacteria) (Supplementary Fig. S1). The highest BE production was obtained in the experiments MP2 and MP3 (0.37 g/L) Fig. 2. with a yield of 0.37 (% gEthanol/gCyanobacteria) (Supplementary Fig. S1). These results were obtained with the following pretreatment conditions S = 0.25, t = 15 min, T = 60 °C and S = 0.25, t = 55 min, T = 40 °C as shown in Supplementary Table S2b. The maximum ethanol concentration (3.02 ± 0.07 g/L) was achieved with MN process at 120 rpm 30 °C.

All extraction samples obtained by MN exhibit a remarkable increase in reducing sugars when compared with control biomass (Supplementary Fig. S2) due to the extraction mixture used for MN, which comprises limonene and ethyl acetate^34^. All extraction samples obtained by MP show a sharp decrease in reducing sugars when compared with control biomass (Supplementary Fig. S2) as long as the extraction mixture used for MP, which comprises water (10 mM ammonium acetate) and ethanol.

For MN a major depletion in the reducing sugars was detected after the first 24 h; in this case, we can assume that hexose sugars are rapidly metabolized since *S. cerevisiae* possess carbon catabolite repression (CCR) with mixed sugars obtained from algal cultures^42^. This result can be useful for the scale up of the process, since we can obtain a higher concentration of BE in short time. Also, it is important to distinguish that these samples exhibited a different performance from the control sample, mainly because in this pretreatment we started at the higher level of reducing sugars, based on the polarity of the solvent used in the extraction process. The maximum content of reducing sugars was obtained under the experiments: MN2 S = 0.25, t = 15 min, T = 60 °C and MN3 S = 0.25, t = 55 min, T = 40 °C Supplementary Table S2b and Supplementary Fig. S2. The highest content of reducing sugars was obtained under the experiments MP8 S = 0.81, t = 55 min, T = 60 °C and MP3 S = 0.25, t = 55 min, T = 40 °C Supplementary Table S2b and Supplementary Fig. S2. In particular we observed an increment of pentose sugars and a decrease of hexose sugars because the hexose sugars are rapidly metabolized as a result of CCR.

Moreover, two sets of conditions, MN and MP, were evaluated as a substrate for LA fermentation by *L*. *acidophilus* ATCC 43121 after the pretreatment with MAE. According to Fig. 4, for MN set, the highest LA concentration was obtained in the experiment MN3, S = 0.25, t = 55 min, T = 40 °C (6.24 ± 0.08 g/L). Regarding the LA yield, the highest value (% gLactic acid/gCyanobacteria) of 0.31 was obtained in the experiment MN3 S = 0.25, t = 55 min, T = 40 °C (Supplementary Fig. S3). Concerning the MP experiments, the highest LA concentration was obtained in the experiment MP3 S = 0.25, t = 55 min, T = 40 °C, (7.26 ± 0.05 g/L), Fig. 4. About the LA yield for MP, the highest value (% gLactic acid/gCyanobacteria) of 0.37 was obtained in the experiment MP3 S = 0.25, t = 55 min, T = 40 °C (Supplementary Fig. S3, Supplementary Table S2b).

As for the assimilation of cyanobacterial biomass by *L. acidophilus* ATCC 43121, we measured the change in reducing sugars content in the time. As shown in Supplementary Fig. S4, all the samples obtained by MN show a remarkable boost in reducing sugars when compared with control biomass. This can be due to the extraction mixture used for MN, which include limonene and ethyl acetate^34^ which are non-polar solvents and as a result of their polarity, we only extract non polar compounds, therefore the concentration of carbohydrates in the biomass increases. Differently from the pretreatment with SF and MP processes, in these results we can observe a major depletion in the reducing sugars after the first 10 h. Consequently, we can assume that hexose sugars are rapidly metabolized^46^. Also, it is important to distinguish that these samples exhibited a different performance of the control sample, mainly because in this pretreatment we started at the higher level of reducing sugars. The maximum content of reducing sugars was obtained under the experiments MN2 S = 0.25, t = 15 min, T = 60 °C and MN3 S = 0.25, t = 55 min, T = 40 °C Supplementary Table S2b and Supplementary Fig. S4.

As it is possible to see in Supplementary Fig. S4, all extraction samples obtained by MP show a big exceptional decrease in reducing sugars when compared with control biomass. This can be due to the extraction mixture used for MP, which comprises water (10 mM ammonium acetate) and ethanol, since glucose has been reported to have a high solubility in ethanol/water mixtures, which is also influenced by temperature^47^, therefore these results can explain the lower content of reducing sugars in the pretreated biomass with MP. Contrasting with the pretreatment with SF processes, in these results we can observe a slight increase in the reducing sugars after the first 24 h. However, after these 24 h, we found a decrease in the reduced sugar contents, that can be due to a reduction in the availability of hexose sugars^46^. The highest content of reducing sugars was obtained under the experiments MP3 S = 0.25, t = 55 min, T = 40 °C and MP4 S = 0.25, t = 55 min, T = 60 °C Supplementary Table S2b and Supplementary Fig. S4. Otherwise from the BE results, the biomass that results from MP process represents a fair option for the LA production in the frame of multi-product biorefinery due to their higher content of LA and the diversity of HVM content^34^. However, in the broad view, the SF pretreatment gives the highest content of LA.

The time course of monosaccharides and protein related to the most representative samples of the *S. cerevisiae* fermentation on MN and MP pretreated *A. platensis* was performed. For, the MN, the experiments MN2 S = 0.25, t = 15 min, T = 60 °C, MN3 S = 0.25, t = 55 min, T = 40 °C and 6 S = 0.81, t = 15 min, T = 60 °C, and for MP, the experiments MP2 S = 0.25, t = 15 min, T = 60 °C, 3 S = 0.25, t = 55 min, T = 40 °C and MP6 S = 0.81, t = 15 min, T = 60 °C, were selected respectively, at two different times based on their high content of BE. Moreover, the profile of monosaccharides showed a higher content of d-glucose (2.48 ± 0.06, 2.31 ± 0.04 g/L) for MN and MP, respectively, in comparison with the other monosaccharides such as d-galactose, d-mannose, d-arabinose, l-fucose and xylose (Fig. 3). Interestingly, the content of d-glucose is variable in terms of pretreatment, i.e. MN has more content than MP, this can be due to the polarity of the solvents used in the extraction processes. Furthermore, in all cases, MN and MP monosaccharides profile fits with the previous reports from the sugar content in *A. platensis*^48,49^. On the other hand, in terms of protein content MP showed a higher content than MN and no changes were observed among the selected samples, (0.38 ± 0.05 g/L), (0.43 ± 0.05 g/L) for MN and MP Fig. 3, respectively.

### Economic analysis

Supplementary Figure S5 summarizes the economic evaluation (also presented in Supplementary Table S3). For the fermenter scale, the values were modified by a base 10 logarithm for easier presentation and calculation. The analyses on BE production experiments are reported in Supplementary Fig. S5a–e, while Supplementary Fig. S5f–j contains those for LA generation. The analysis presented corresponds to five variables with five levels each which generates a total of 3125 combinations. For this reason, all the graphs in Supplementary Fig. S5 are arranged in five vertical sets which correspond to the five levels for the variable being analyzed, while the dispersion in each set corresponds to the impact of the four remaining variables.

The range of calculated costs (per gram of product) for BE production in fermentation was from US$ 0.07 to 1125.17 and for LA from US$ 0.02 to 351.4. These production costs were the result from the 3125 combinations and covered a wide range of scenarios and combinations of variables. The conditions for the lowest production costs calculated were the optimal scenarios tested: the fermenter scale was 10,000 L and thus the fermenter cost was 1000 USD with a fermentation time of 12 h and cyanobacterial biomass cost of 0.01 USD. Additionally, for LA the production titer was at 38.68 g/L while for BE the value was 12.08 g/L. It is important to consider that these conditions might be above current real values, but they were key to show the deep impact of improving current conditions on production costs (Supplementary Table S3). The major impact on the length of the range of CoG/g for both products was associated to the contribution of the productions scale (1–10,000 L).

It is important to mention that the focus of this economic analysis was to elucidate the possible extra costs that could be generated from producing of BE and LA thorough fermentative processes, for this reason, only the costs associated with fermentation were considered here as reported elsewhere^50^. The consideration of only production associated costs allows to focus on the contributions performed by the process developed here without losing the focus on other costs^51,52^. This means that costs associated with collection method, commercialization, storage and distribution were not considered because were out of scope of this study.

In order to analyze the generated data, a linear regression model was calculated to determine the linear coefficients for the contribution for each parameter (Table 1). Together, Supplementary Fig. S5 results and the linear model (Table 1) allowed to reach some interesting insights on both commodity chemicals production. The linear model showed the contribution of each single parameter in a simultaneous calculation. For the results obtained, each coefficient represents the slope that the given parameter generates on the CoG/g.

**Table 1:** Linear regression results for the Monte Carlo simulations. Data shows coefficients for the regression equation. All analyzed parameters were included, and their coefficient determine the impact they each have on the CoG/g for each product (BE or LA).

Regression components^a^	Regression coefficients	
	Ethanol production	Lactic acid production
Intercept	− 8.06	− 2.52
Fermenter scale^b^	− 18.35^c^	− 5.73^c^
Fermenter cost^b^	16.67^c^	5.21^c^
Ethanol titer	− 3.22^c^	N/A
Lactic acid titer	N/A	− 0.31^c^
Fermentation time	0.21^c^	0.06^c^
Biomass cost^b^	0.13	0.04

^a^Regression with the form CoG/g = β0 + β1 × Log_10_ (Fermenter Scale in L) + β2 × Log_10_ (Fermenter Cost in US $) + β3 × (Ethanol or Lactic Acid Titer in g/L) + β4 × (Fermentation Time in h) + β5 ×Log_10_ (Biomass Cost in US $).

^b^Input for this parameters is performed in logarithmic scale (base 10).

^c^Statistically significant to α = 0.01.

## Discussion

The huge increase of CO_2_ concentration in the atmosphere, due to the burning of fossil resources and several production processes and human activities, resulting in global warming and climate change^53,54^ and threating the survival of many species and global ecosystem’s health^55,56^, represents an important driver of the increasing attention on cyanobacteria and their use for CO_2_ biological sequestration^57,58^.

As far as the use of *A. platensis* for CO_2_ sequestration, this cyanobacterium belongs to the alkaliphilic cyanobacteria with selective growth conditions such as high pH and alkalinity^58^. These conditions prevented overgrowth of other organisms even in outdoor open-pond cultivation and allowed relatively easy quality control^59–61^. Among alkaliphilic cyanobacteria, *A. platensis* was the most commercially produced genus because of its content of HVM^62,63^. Moreover, the alkaline medium of *A. platensis* was suitable for CO_2_ recovery through chemical absorption^64,65^. The biggest advantage was the high alkalinity that could trap CO_2_ in a more efficient way. For example, dissolved inorganic carbon (DIC) optimum concentration was in the range of 0.01–0.1 mol/L in freshwater species like *Scenedesmus* sp.^66^ while it is 0.1–0.4 mol/L for *A. platensis*^65^. With this high alkalinity, the alkaliphilic algal medium can absorb much more CO_2_ into the same amount of liquid without pH change due to the buffer function of DIC.

In addition, because of metabolites richness, the members of the *Arthrospira* genus are remarkable sources for HVM^62^. For example, in 2002, FDA (Food and Drug Administration from the US) classified *Arthrospira *(Formerly *Spirulina*) preparations as GRAS (Generally Regarded As Safe). Later, in 2011 the United States Pharmacopeia, assigned a Class A safety rating for *A. maxima* and *A. platensis*, thereby permitting the admission of these cyanobacteria as a dietary supplement ingredient. Also, it has been reported the safety evaluations of bioactivities of an *A. platensis* diet in a murine model with favorable results^67^. Furthermore, in 2011 FAO (Food and Agriculture Organization) announced that *Arthrospira* was an interesting food due to its high contents of iron and protein, since its use can reduce malnutrition of the habitants of low-income countries. Therefore, the use of *A. platensis* as source of HVM is very broad and easily can extrapolated to the pharma and food industry as diet supplementary food^68,69^.

Similarly, the photosynthetic cyanobacterium *A. platensis* have received much attention for its great capacity of HVM production. As matter of fact, in previous experiments, pretreatments consisting of supercritical fluid extraction (SF) and microwave assisted extraction with non-polar (MN) and polar solvents (MP), carried out on *A. platensis* allowed to obtain several bioactive metabolites^34,35^.

Based on the above, in this work residual biomass of *A. platensis* after extraction of HVM by SF, MN or MP was tested as renewable feedstock for production of BE and LA for a complete valorization of all cell components in an integrated biorefinery with the aim to join the production of specialties (HVM) and bulk products (BE, LA) towards to zero biomass waste.

The adoption of SF and MP/MN, as pretreatments for HVM extraction from *A. platensis* was related^70,71^ to several advantages of these processes: they use alternative solvents, produce co-products instead of waste, and reduce unit operations^70,72,73^. Moreover, it should also be noted that while SF is thought to be very energy intensive, several reports showed that SF was fairly competitive with conventional extraction methods^74–76^. A life cycle assessment (LCA) showed that SF of wet algae might be less energy demanding than hexane extraction on either dry or wet algae^77^. Also, with MP/MN processes, it has been demonstrated that using 5 min of 1021 W microwave irradiation would contribute < 1% to the energy input, while increasing lipid extraction threefold in comparison with a control in the extraction of lipids from *Nannochloropsis oculata* slurries (77% water content) by using ethanol and hexane as extraction solvents over a range of time and energy levels^78^.

As for the use of SF as a pretreatment, it has been previously reported for microalgae/cyanobacteria biomass. For example, the cell wall of *Chlorococcum* sp. was ruptured due to the temperature and pressure required for the process, and enhanced the release of several polysaccharides embedded in the cell wall^79^.

Actually, few studies have been reported on the use of *A. platensis* biomass for the production of BE whilst there are not studies on BE production from *A. platensis* depleted biomass using *S. cerevisiae*. For example, an interesting approach by Markou et al.^80^ was the saccharification of the carbohydrate enriched biomass with this approach the highest BE production of 2.03 of g/L was obtained from the hydrolysate by nitric acid (0.5 N). A direct conversion of *A. platensis* to BE mediated by the use of lysozyme and a recombinant amylase-expressing yeast strain has produced a concentration of BE of 6.5 g/L^81^. Also, the technical feasibility of the BE production from *A. platensis* has been evaluated with a percentage of BE among 0.85–1% (w/w) and by the measurement of the variation of BE percentage in the final solution according to the variation of air-drying time using three different air-drying times (1 day, 2 days and 3 days air drying) by Hossain et al.^82^.

Additionally, there are several studies on production of BE using other algal cultures. It has been reported the production of BE from an hydrolysate of *Chlorella* sp. biomass (1.5% of sulfuric acid at 117 °C for 20 min) with a concentration of 5.62 ± 0.16 g/L of BE by the fermentation of *S. cerevisiae* TISTR 5339^83^. A microalgae mixed culture (obtained from a freshwater area in Osku located in northwest of Iran) was pretreated with an enzymatic mixture (B-glucosidase, a-amylase and amyloglucosidase) and evaluated to produce BE by fermentation with *S. cerevisiae* (ATCC 7921), the obtained results showed that the highest content of BE was 6.41 g/L^84^. Biomass from *Porphyridium cruentum* (KMMCC-1061) was subjected to an enzymatic hydrolysis with pectinase and cellulase enzymes and therefore was used as carbon source for a fermentation process with *S. cerevisiae* KCTC 7906, the results showed that the highest content of BE was 0.65 g/L^85^. Based on this, in comparison with *P. cruentum* cultures from Kim et al.^85^, the values of BE concentrations we obtained are higher but in comparison with Ngamsirisomsakul et al.^83^ our results are lower. This behavior can be explained by the chemistry of the pretreatment method as if water was used as solvent, this can change the monosaccharides composition in the depleted cyanobacterial biomass. This is shown in Supplementary Fig. S2 where the control has a lower content of reducing sugars than the pretreated samples.

To the best of our knowledge, there are not studies of LA production from *A. platensis* depleted biomass. However, in this frame, some studies based on other algal cultures were reported. The use of lyophilized biomass of the *A. platensis* as the substrate for LA fermentation by the probiotic bacterium *Lactobacillus plantarum* ATCC 8014 has been reported achieving a LA concentration of 3.7 g/L^86^. Moreover, algal carcass (AC) a low-value byproduct of algae after its conversion to biodiesel was used to ferment algal carcass media (ACM), including 2% ACM with 1.9% glucose and 2 g/L amino acid mixture (ACM-GA) with *Lactobacillus delbrueckii* ssp. bulgaricus ATCC 11842. The results showed that the highest content of LA was 3.31 g/L^87^. Furthermore, biomass from *Enteromorpha prolifera* was hydrolyzed with 0.5 M sulfuric acid at 120 °C for 2 h, and therefore was used as carbon source for a fermentation process with *Lactobacillus rhamnosus*, the results showed that the highest content of LA was 4.3 g/L^88^. Also, biomass from *Gelidium amansii* was hydrolyzed with 3% v/v sulfuric acid at 140 °C for 5 min, therefore the hydrolysate was fermented with *L. rhamnosus* and the highest content of LA was 12.5 g/L^89^.

In reference to this information, we have a higher content of LA than the results from other algal cultures such as algal carcass and *Enteromorpha prolifera,* but we have a lower content in comparison with the use of *Gelidum amansii* biomass. These results can be due to the different composition of algal biomass, since they are part of a big biological group with several differences between them. Furthermore, *L. acidophilus* is an obligated homo-fermenter and is among the least oxygen-tolerant lactobacilli^90^. Also, different transporters that allow the uptake of several carbohydrates (e.g. glucose, fructose, and trehalose) have been identified and characterized. This catabolic machinery is highly regulated at the transcription level, and suggests that the *L. acidophilus* transcriptome is flexible, dynamic, and designed for efficient carbohydrate utilization. Furthermore, *L. acidophilus* seems to adapt its metabolic machinery depending on the available carbohydrate sources^43,91^, and it is a suitable microorganism for LA production and biorefinery with cyanobacterial biomass.

Additionally, according to our previous reports^35^, similarly to the BE results, the SF experiment SF8 CS = 11 g/min, P = 450 bar, Supplementary Table S2a offers the best option in terms of suitability for the multi-product biorefinery due to the highest extraction yield (7.48% w/w), while for MN, the LA content is lower than the results from SF pretreatment due to the differences between the extraction processes in terms of the types of solvents (Supercritical carbon dioxide vs limonene, ethyl acetate, hexane i.e.). According to our previous reports^34^, experiment MN3 S = 0.25, t = 55 min, T = 40 °C represents a good option for the multi-product biorefinery due to its good extraction yield (4.67% w/w) of bioactive compounds and its specific conditions Supplementary Table S2b, while the contents of LA in MP pretreatments were lower than the SF and MN values.

The results of our economic analysis showed that process scale had the largest impact on the cost, this impact has been reported before^92^ as one of the major contributors of a wide range of production costs. Alternatively, the use of the best production titers obtained from the fermentation experiments (3.02 g/L for BE and 9.67 g/L for LA) and their range of analysis (four times above and below the base value) generated the large difference of the production costs for each commodity chemical. Production titer has been reported^93^ to be one of the major driving forces for causing production costs variations, but comparatively, as the initial or base fermentation titer increased (as for LA) the overall effect is reduced. This is seen by the width of the range for the CoG/g for LA, opposite to the range of CoG/g for BE which spans for more than US$ 1000. As a summary, the range of the CoG/g was a consequence, mainly, from the fermentation scales analyzed, while the different ranges for each product was associated to the different production titers for BE and LA.

Regardless of the product (BE or LA), the ranking of the impact was (in order of importance): fermenter scale, fermenter cost, production titer, and fermentation time. The cost of the cyanobacterial biomass resulted as not statistically significant. This means that regardless of the scale, the cost of the fermenter to use or the production achieved was not affected by the cost of generating the cyanobacterial biomass. This is particularly significant as the use of microwave and solvent-assisted extractions can have different impacts by involving expensive unit operations or materials (in the case of solvent extraction) and have no significant impact on the cost of the fermentation. Furthermore, our results indicated that both meticulous rethinking of process technology and adaptation of the process are compulsory. Also, an exhaustive valorization of all elements in a biorefinery will promote the transition towards to an optimization of the costs of a multi-product biorefinery. Along these lines, in the present work we introduced an innovative approach with green extraction technologies (for specialty products or HVM) coupled to fermentation processes (for bulk products) to reduce the global costs of a multi-product biorefinery.

This study analyzed the technical and economic potential of a multi-product biorefinery from *A. platensis* biomass based on the production of bulk commodities and HVM. BE and LA possess a well-developed market. The first one can be used as a third-generation biofuel and the former possess a broader range of applications in the food, chemical and bioplastics industries. The main drawback for the bulk production of these bioproducts are the carbon source and their cultivation. To overcome these drawbacks, a multi-product biorefinery can accelerate their production at industrial level.

The cyanobacterial biomass has proven to resolve the feedstock problems with first- and second-generation biofuels. Also, the present study has demonstrated that the biomass resulting from SF or MP/MN process could be used as carbon source for BE and LA production because its residual carbohydrates were readily available for fermentation. For BE, MN pretreatment showed the highest content (3.02 ± 0.07 g/L), while for LA, SF pretreatment showed the highest content (9.67 ± 0.05). Additionally, from the economic evaluation, the cost of cyanobacterial biomass generation was not statistically significant for the BE and LA production costs. Green extraction process to produce HVM and the subsequent use of the depleted biomass confirmed the suitability for the development of multi-product biorefinery with *A. platensis* as a feedstock. To the best of our knowledge, this is the first study in which *A. platensis* biomass pretreated by SF and MP/MN was used as feedstock for BE and LA production.

## Material and methods

### Material

Ammonium sulfate ((NH_4_)_2_SO_4_), potassium phosphate dibasic (K_2_HPO_4_), potassium phosphate monobasic (KH_2_PO_4_), zinc chloride (ZnCl_2_), magnesium sulfate (MgSO_4_), 3,5-Dinitrosalicylic acid (DNS), sulfuric acid (H_2_SO_4_), potassium hydroxide (KOH), glucose (C_6_H_12_O_6_), bradford reagent and yeast extract were obtained from Sigma Chemical Co., (St. Louis, MO, USA). Bacto peptone (an enzymatic hydrolyzate of bovine and porcine animal proteins) and malt extract were obtained from BD Biosciences (Heidelberg, Germany). MRS broth from Oxoid Ltd (Basingstoke, United Kingdom).

### Cyanobacteria cultivation

The cyanobacteria strain *Arthrospira platensis* used throughout this work was cultivated as reported in Esquivel-Hernandez et al.^34,35^. Briefly, *A. platensis* was grown for 45 days in open raceway ponds with modified Jourdan composed of (g/L): NaHCO_3_ (5.88), Na_3_PO_4_ (0.16), NaNO_3_ (0.92), MgSO_4_·7H_2_O (7.07), FeSO_4_ (0.004) and NaCl (2). The geographical location of the ponds was 20″ 1410″ N, 103″ 3510″ W. The biomass was harvested with a mesh, air dried to 20% moisture and stored under dry and dark conditions until analysis. The complete frame of multi-product biorefinery appears in Fig. 1.

### Extraction of high value metabolites from cyanobacteria by supercritical fluid extraction (SF)

Supercritical fluid extraction (SF) of HVM from *A. platensis* cells was performed according to our previous work^35^. Briefly, all extractions were carried out using a 100 mL extraction cell (Thar SFC SFE 100, Waters Corp., Milford, MA, USA) with25 g/min CO_2_ flow and ethanol as co-solvent. A Plackett–Burman design that is reported in Supplementary Table S2a was used for the six experimental factors generating 12 experimental conditions tested with triplicates carried out in randomized run order. Each factor was tested at two different experimental levels: Co-solvent (CX) (g/min 4, 11); pressure (P) (bar 150, 450); static extraction (SX) (min 5, 15); dynamic extraction (DX) (min 25, 55); temperature (T) (°C 40, 60); and, dispersant (Di) (g 0, 35).

The results of each response variable and their statistical significance were analyzed with ANOVA (*p* > 0.05), using the statistical package Minitab 16 (State College, PA, USA). The cyanobacterial residual biomass was dried at 60 °C in an oven to eliminate impurities that were generated in the course of the SF process before fermentation process.

### Extraction of high value metabolites from cyanobacteria by microwave-assisted extraction (MP/MN)

Microwave-assisted extraction of HVM from *A. platensis* cells was performed according to our previous work^34^. Briefly, these experiments were carried out in a microwave-assisted extraction equipment MARS 5 (CEM Corporation, Matthews, USA) with a 100 mL extraction vessel Green-Chem. The extraction vessel was built with PFA Teflon. All extractions were done at 400 W power and 1 bar. For each experiment, the extraction vessel was filled with a 0.14 ratio of dry weight biomass/solvent (w/v). The solvent ratio was defined as a v/v ratio (v/v; 0.25, 0.81) of the combination of two types of solvents, polar solvents MP (Ammonium acetate 10 mM and ethanol) and non-polar solvents MN i.e., Limonene (1-methyl-4-(1-methyle thenyl)-cyclohexene) and ethyl acetate with a 2^k^ (Standard factorial design) based on three experimental factors generating 8 experimental conditions (1–8) tested with triplicates carried out in randomized run order with the experimental matrix design reported in Supplementary Table S2b. The results of each response variable and their statistical significance were analyzed with ANOVA (*p* > 0.05), using the statistical package Minitab 16 (State College, PA, USA). The cyanobacterial residual biomass was dried at 60 °C in an oven in order to eliminate impurities that were generated throughout the MP/MN before fermentation process.

### Analysis of sugar content of residual cyanobacterial biomass

In order to determine the cyanobacterial biomass composition, the samples of SF, MN and MP pretreatments with highest yields for BE and LA, respectively (Supplementary Table S2; Supplementary Figs. S1, S3) were selected and subjected to acid hydrolysis. 5 g of lyophilized cyanobacterial depleted biomass were mixed with sulfuric acid to reach at a final acid concentration of 0.1% (v/v). The resulting slurries were then autoclaved at 121 °C for 20 min. After hydrolysis, samples were cooled to room temperature, centrifuged at 4 °C and 9000*g* for 20 min and the supernatant containing the released sugars was collected as acidic hydrolysate. The sugar content and composition were then measured by HPLC method.

### *Saccharomyces cerevisiae* inoculum and medium preparation for ethanol production

The yeast strain adopted for BE fermentation was *S. cerevisiae LPB-287* selected in our previous work^94^. The yeast was inoculated in three 250 mL flasks containing 100 mL of YM broth composed of (g/L): glucose (10), peptone (5), malt extract (3), yeast extract (3). The medium was autoclaved at 121 °C, 30 psi during 30 min. The cultivation was carried out at 30 °C with 120 rpm for 24 h and. After 24 h, cells were separated by centrifugation at 10,000 rpm for 15 min and washed three times with sterilized water. BE production was carried out with the medium proposed for *A. platensis*^80^ with slight changes composed of (g/L): (NH_4_)_2_SO_4_ (2), K_2_HPO_4_ (1), KH_2_PO_4_ (1), ZnSO_4_ (0.2), MgSO_4_ (0.2) and yeast extract (2). Fermentation was carried out with the strain *S. cerevisiae LPB-287* in 250 mL Erlenmeyer flasks. 10 g/L concentration of cyanobacterial biomass was prepared (for all samples from SF and MP/MN) and added to 50 mL of sterilized fermentation medium. 5 mL of yeast culture was harvested in the exponential phase and used to inoculate each fermentation medium aseptically. Flasks were incubated at 30 °C under 120 rpm during 81 h. Samples everywhere withdrawn each 24 h.

### *Lactobacillus acidophilus* inoculum and medium preparation for lactic acid production

*Lactobacillus acidophilus* ATCC 43121, which was selected in our previous work^95^, was used for LA production. De Man, Rogosa and Sharpe broth (MRS) was used as the growth medium for the inoculum. The medium was autoclaved at 121 °C, 30 psi during 30 min. The strain was inoculated in three 250 mL flasks containing 100 mL of MRS broth and cultivated by static incubation for 24 h and 37 °C. After 24 h, cells were collected by centrifugation at 10,000 rpm for 15 min and washed three times with sterilized water. The medium for the LA production was prepared according to the medium proposed by  Nguyen et al.^96^ with slight changes. The composition in (g/L) was as follows: Peptone (3), yeast extract (3). The medium was autoclaved at 121 °C, 30 psi during 30 min. Lactic fermentations were carried out with the strain *L. acidophilus* ATCC 43121 in 250 mL Erlenmeyer flasks. 20 g/L concentration of cyanobacteria substrates were prepared (for all samples from SF and MP/MN) and added to 50 mL of sterilized fermentation medium. 5 mL of inoculum were used to inoculate each flask. Flasks were incubated at 37 °C under 120 rpm during 81 h. Samples everywhere withdrawn each 24 h.

### Analytical methods

Reducing sugars concentration was analyzed using the dinitrosalicylic (DNS) method^97^. The absorbance was measured at 575 nm, using Novaspec II spectrophotometer.

BE was determined using commercial kits (K-ETOH) from Megazyme International (Wicklow, Ireland). The calculations were done with Megazyme Mega-Calc and expressed as g ethanol/L. LA was determined using commercial kits (KDLATE) from Megazyme International (Wicklow, Ireland). The calculations were done with Megazyme Mega-Calc and expressed as g lactic acid/L. The monomeric sugars content was determined by high performance anion-exchange chromatography (HPAEC; DX-300 series chromatography system, Dionex, USA). The effluent was monitored with pulsed amperometric detection detector (PAD, Dionex, CA, USA). Filtered samples (10 μL) were injected into a CarboPac PA-1 anion-exchange column (0.4 × 250 mm, Dionex, CA, USA) that was pre-equilibrated with 18 mM KOH. The isocratic method was employed with 18 mM KOH at a flow rate of 1.0 mL/min in 20 min. Protein content was determined by the Bradford method with OD measured at 595 nm^98^. Each measurement was repeated at least three times and averaged.

### Model set-up

The model presented in this work was constructed using Biosolve Process (Biopharm Services, Buckinghamshire, UK). Briefly, to construct a bioprocess model three main areas are required^99^, not necessarily in this order: (1) design of the production scenarios and target output, (2) the unit operations and process parameters and (3) the economic datasets. BE and LA production processes were both modelled as they were conducted in separate economic activities, microwave- and solvent-assisted extractions. This is a preliminary approach to help future decision-making for future endeavors in the production of these commodity chemicals.

Different scenarios were presented so as to have a certain flexibility. Therefore, a set of five variables (Fermenter scale, fermenter cost, BE/LA production, fermentation time and cyanobacterial biomass cost) were selected at five levels each) and their combinations were evaluated corresponding to 3125 scenarios as shown in Table 2. The second model construction component considered BE or LA fermentation, which means that process parameters are fermentation related. Briefly, fermentation conditions, such as fermentation time, media and production titer (in g/L) were retained as experimentally determined in previous sections. To fulfil the economic dataset, which will ultimately give the economic results, costs for material and consumables were either collected from Sigma-Aldrich at their largest available presentation (least expensive presentation, but without underestimating potential costs) or from Biosolve Process database. Equipment costs (fermenter) were considered (Supplementary Table S3). Labor was fixed at 15% of the production costs as it has been reported by Heinzle et al.^100^. Last, as this is a preliminary study, there is not a fixed potential cost or price for the acquisition of cyanobacterial biomass, so it was decided to use a range from US$ 0.01 to 100/kg. As a result, from model calculations and analyses, the cost of goods per gram of product (CoG/g) is recorded. All data used for model construction is included in Supplementary Material.

**Table 2:** Scenarios evaluated in the economic analysis. For regression analysis and figures the fermenter scale, fermenter cost and biomass cost were changed to a logarithmic (base 10) scale.

Fermenter scale (L)	Fermenter cost (US $)	Ethanol production (g/L)	Lactic acid production (g/L)	Fermentation time (h)	Biomass cost (US $)
1	1000	0.755	2.4175	12	0.01
10	5000	1.51	4.835	24	0.1
100	10,000	3.02	9.67	36	1
1000	50,000	6.04	19.34	72	10
10,000	100,000	12.08	38.68	144	100

After the completion of the model construction, Table 2 scenarios were input into the model and their results registered. Additionally, with the results generated a linear regression for the five independent and one dependent variables was calculated to determine the coefficients for each variable^101^. This in turn, depending on the value of each coefficient, will determine the impact each variable has on the production costs.

## Supplementary Information

Supplementary Information.
