# Supplementary material for: Multi-product biorefinery from Arthrospira platensis biomass as feedstock for bioethanol and lactic acid production
Source: Sci Rep. 2021 Sep 29;11:19309. doi: 10.1038/s41598-021-97803-5 (PMC8481326; doi:10.1038/s41598-021-97803-5)
Supplement: Supplementary file 1 — Supplementary Information. [file 41598_2021_97803_MOESM1_ESM.docx]

**Multi-Product biorefinery from *Arthrospira platensis* biomass as feedstock for bioethanol and lactic acid production**

Diego A. Esquivel-Hernández^1,2,3^, Anna Pennacchio^4^, Mario A. Torres-Acosta^5^, Roberto Parra-Saldívar^1^, Luciana Porto de Souza Vandenberghe^6^ and Vincenza Faraco^4^*

^1^ Escuela de Ingenieria y Ciencias, Tecnologico de Monterrey, Campus Monterrey, Ave. Eugenio Garza Sada 2501, Monterrey, N.L. 64849, Mexico

^2^Departamento de Microbiologia Molecular, Instituto de Biotecnologia, Universidad Nacional Autónoma de México, Ave. Universidad 2001, Cuernavaca, Morelos, 62210, Mexico

^3^Departamento de Biología Celular, Facultad de Ciencias, Universidad Nacional Autónoma de México, Circuito Exterior s/n, 04510, Ciudad de México, México

^4^Department of Chemical Sciences, University of Naples “Federico II”, Complesso Universitario Monte S. Angelo, Via Cintia 4, 80126 Naples, Italy

^5^ The Advance Centre for Biochemical Engineering, Department of Biochemical Engineering, University College London, London, WC1E 6BT, United Kingdom

^6^ Department of Bioprocess Engineering and Biotechnology, Federal University of Paraná Coronel Francisco H. dos Santos Avenue, 210, 81531-980 Curitiba, Brazil

***Corresponding author:** Vincenza Faraco **Email:** vfaraco@unina.it

**Supplementary Material**

**Fig. S1** Time course of yield of BE in the fermentation process by *Saccharomyces cerevisiae LPB-287* with depleted cyanobacteria biomass from SF, MN and MP pretreatments. *(factor(Ext) refers to the type of pretreatment respectively, n=3, time of fermentation 90h)

**Fig. S2** Time course of reducing sugar concentration measured by DNS method in the fermentation process by *Saccharomyces cerevisiae LPB-287* with cyanobacteria from SF, MN and MP pretreatments. *(factor(Ext) refers to the type of pretreatment, respectively, n=3, CL is a positive control, time of fermentation 90h)

**Fig. S3** Time course of yield of LA in the fermentation process by *Lactobacillus acidophilus* ATCC 43121 with depleted cyanobacteria biomass from SF, MN and MP pretreatments. *(factor(Ext) refers to the type of pretreatment respectively, n=3, time of fermentation 90h)

**Fig. S4** Time course of reducing sugar concentration measured by DNS method in the fermentation process by *Lactobacillus acidophilus* ATCC 43121 with cyanobacteria from SF, MN and MP pretreatments. *(factor(Ext) refers to the type of pretreatment, respectively, n=3, CL is a positive control, time of fermentation 90h)


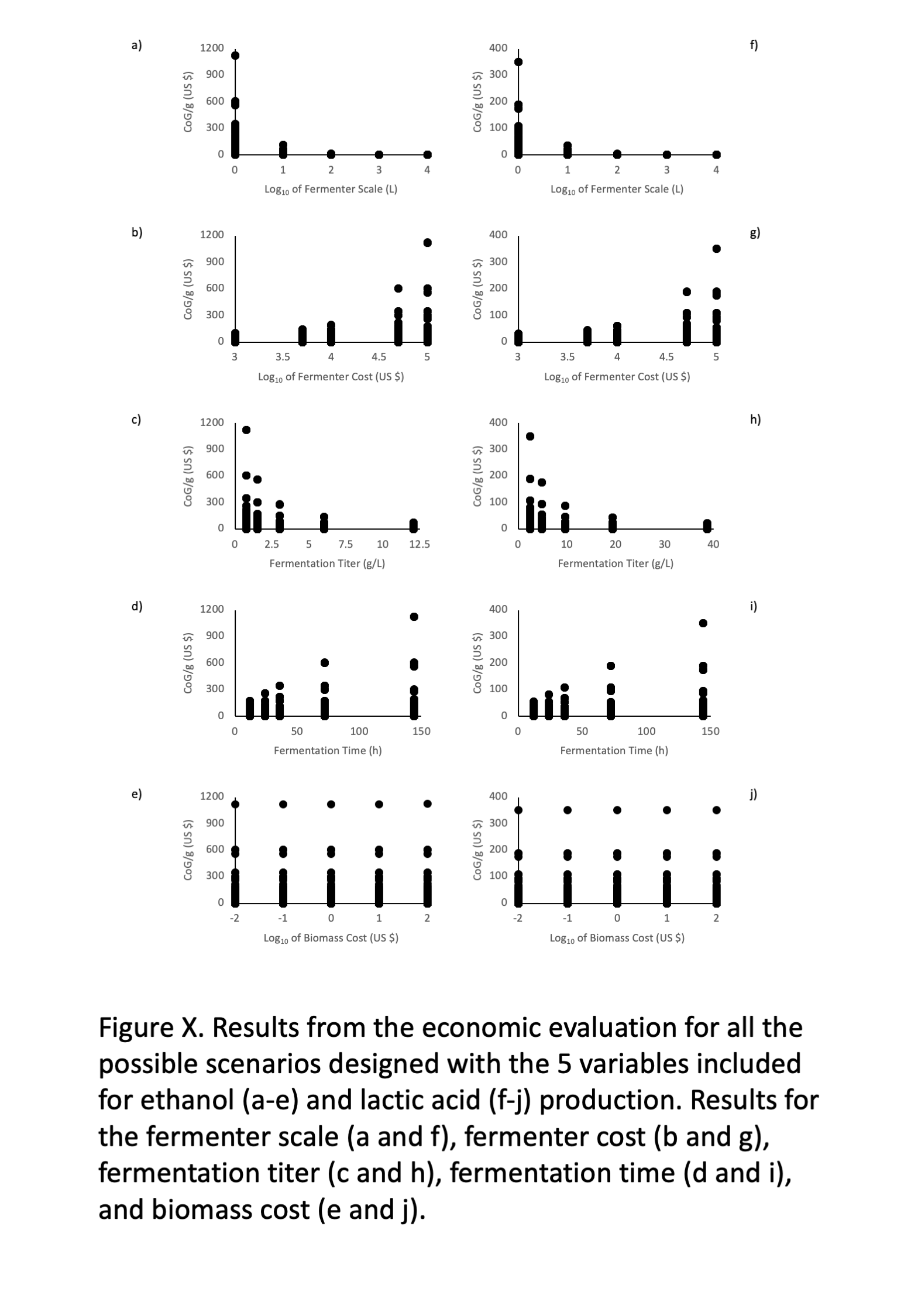


**Fig. S5** Results from the economic evaluation for all the possible scenarios designed with the 5 variables included for ethanol (a-e) and lactic acid (f-j) production. Results for the fermenter scale (a and f), fermenter cost (b and g), fermentation titer (c and h), fermentation time (d and i), and biomass cost (e and j)

**Table S1.** Chemical composition of selected extracts of *A. platensis* biomass obtained by green technologies SF and MAE (MP, MN).

| Green | High-Value Metabolites | | | | |
| --- | --- | --- | --- | --- | --- |
| Technology* |  |  |  |  |  |
| SF-Run | α-tocopherol  μg/g | γ-linolenic acid  mg/g | β-carotene  μg/g | Lutein  μg/g | C-phycocyanin  μg/g |
| SF2 | 5.49 ± 0.10 | 9.95 ± 0.21 | 524.46 ± 0.10 | 1.44 ± 0.10 | -- |
| MP-Run |  |  |  |  |  |
| MP2 | -- | -- | -- | -- | 2.28 ± 0.10 |
| MN-Run |  |  |  |  |  |
| MN4 | 37.86 ± 0.78 | 6.80 ± 0.21 | 123.64 ± 1.45 | -- | -- |

*Run refers to experimental conditions described in Table S2. These data were taken from our previously published articles^1,2^.

**Table S2.** Experimental designs for green extraction processes.

1. Supercritical Fluid Extraction (SF)

| Run | CS  (g/min) | P  (bar) | SE  (min) | DE (min) | T (°C) | DS  (g) |
| --- | --- | --- | --- | --- | --- | --- |
| SF1 | 4 | 450 | 5 | 25 | 40 | 35 |
| SF2 | 11 | 450 | 15 | 25 | 60 | 35 |
| SF3 | 11 | 450 | 5 | 55 | 60 | 0 |
| SF4 | 4 | 150 | 5 | 55 | 60 | 35 |
| SF5 | 11 | 150 | 15 | 55 | 40 | 35 |
| SF6 | 4 | 150 | 15 | 55 | 60 | 0 |
| SF7 | 4 | 150 | 5 | 25 | 40 | 0 |
| SF8 | 11 | 450 | 5 | 55 | 40 | 0 |
| SF9 | 11 | 150 | 15 | 25 | 40 | 0 |
| SF10 | 4 | 450 | 15 | 55 | 40 | 35 |
| SF11 | 11 | 150 | 5 | 25 | 60 | 35 |
| SF12 | 4 | 450 | 15 | 25 | 60 | 0 |

Co-solvent (CX), pressure (P), static extraction (SX), dynamic extraction (DX), temperature (T), dispersant (Di).

1. Microwave assisted Extraction MAE with polar (MP) and non-polar solvents (MN).

| Run | Run | S^1^  (v/v) | t  (min) | T  (°C) |
| --- | --- | --- | --- | --- |
| MP1 | MN1 | 0.25 | 15 | 40 |
| MP2 | MN2 | 0.25 | 15 | 60 |
| MP3 | MN3 | 0.25 | 55 | 40 |
| MP4 | MN4 | 0.25 | 55 | 60 |
| MP5 | MN5 | 0.81 | 15 | 40 |
| MP6 | MN6 | 0.81 | 15 | 60 |
| MP7 | MN7 | 0.81 | 55 | 40 |
| MP8 | MN8 | 0.81 | 55 | 60 |

^1^ Solvent ratio refers to ammonium acetate 10 mM and ethanol (v/v) for MP and limonene and ethyl acetate for MN, Time (Ti), and Temperature (Te).

**Table S3** Variable set for the model set-up

| Cost Category | Item | Cost Data |
| --- | --- | --- |
| Equipment |  |  |
|  | Fermenter (all costs were matched with every scale for multiple scenarios evaluation) | Scales (L): Variables according to Table 2. Cost (US$ x 10^3^): Variable according to Table 2. |
|  |  |  |
| Materials |  |  |
|  | For ethanol (g/L) | Cost in US$ *per* kg |
|  | (NH_4_)_2_SO_4_ (2) | 18.01 |
|  | K_2_HPO_4_ (1) | 110.69 |
|  | KH_2_PO_4_ (1) | 32.88 |
|  | ZnSO_4_·7H_2_O (0.35624) | 108.10 |
|  | MgSO_4_ (0.2) | 36.88 |
|  | Yeast Extract (2) | 55.94 |
|  | Biomass (10) | 1.00 |
|  |  |  |
|  | For lactic Acid |  |
|  | Peptone (3) | 51.20 |
|  | Yeast Extract (3) | 55.94 |
|  | Biomass (20) | 1.00 |
|  |  |  |
| Consumables |  |  |
|  | Fermenter filters | Calculated using Biosolve own regression:  $Cost \left[ US\$ \right]=0.3058*Vessel Volume [L]+43.334$ |
|  |  |  |
| Labor |  |  |
|  | Labor | Fixed at 15% of the CoG/g at every simulation |

**References**

1 Esquivel-Hernández, D. A. *et al.* Effect of Supercritical Carbon Dioxide Extraction Parameters on the Biological Activities and Metabolites Present in Extracts from Arthrospira platensis. *Marine drugs* **15**, 174 (2017).

2 Esquivel-Hernández, D. A. *et al.* Advancement of green process through microwave-assisted extraction of bioactive metabolites from Arthrospira Platensis and bioactivity evaluation. *Bioresource technology* **224**, 618-629 (2017).
